# Bibliographical Notices

**Published:** 1844-09-21

**Authors:** 


### BIBLIOGRAPHICAL NOTICES.

*A Practical Treatise on Organic Diseases of the Uterus: Being the* Prize Essay *to which the Medical Society of London awarded the Forthergillian Gold Medal for* 1843. By John C. W. Lever, M. D., Member of the Royal College of Physicians, London, &c., &c. Octavo, pp. 240. London, 1843.

This is a useful book, containing much practical matter in reference to an important class of diseases. The author has enjoyed unusual advantages for studying the subjects on which he has written, having, as one of the physicians of Guy's Hospital, the charge of from one to two hundred patients, weekly, labouring under functional and organic diseases of the womb. The fruits of this large experience are presented to us in the volume before us, methodically arranged and clearly expressed.

Diseases of the uterus are arranged by the author into

"two grand divisions." The one termed "Functional," including those diseases dependent on deviation from the normal action of any part of the organization, is indicated by certain symptoms during life, but which after death are not found to be associated with any appreciable structural change. The second division, viz., Structural or Organic Diseases, includes all diseases in which there is any alteration or lesion of the organ."

In the introductory part of his work, Dr. Lever advances several points for consideration and inquiry, of very great importance.

"Firstly: What is the relative proportion of functional to organic or structural changes?" In answer to this it is stated that of 1388 patients, 905 laboured under functional, and 483 under organic diseases.

Secondly: "Are married or unmarried women more liable to structural changes?"

"Of 95 females affected with polypus, carcinoma, fungoid disease, and hard tumour, 89 were married and 6 were single, the married amounting to 93.68 per cent., to 6.3 per cent.: thus it would appear that marriage tends to favour the development of structural change."

Thirdly: "Does the diathesis accompanying organic disease impair the faculty of conception?"

"Of the 89 married, previously referred to, 8 have never conceived. Thus the proportion of sterile women was 0.9 per cent., a very small proportion; for it is found that 1-20th or 5 per cent. of married woman are wholly unprolific. The child bearing women amount to 91.09 per cent."

After pointing out all the various methods of examination to be pursued in the investigation of uterine disorders, and "the general symptoms of organic disease," the remainder and principal part of the work, is divided into three *parts*, which treat,

1st. Of inflammation of the uterus (acute and chronic.)

2d. Specific diseases, including polypus, hard tumours and strumous tubercles, and those from impure sexual congress.

3d. Malignant diseases; as cauliflower excrescence, corroding ulcer, melanosis, and cancer or carcinoma of the uterus.

Acute inflammation of the uterus in the unimpregnated state, the author has found to occur from suppressed menstruation, or, from the use of astringent injections to cure leucorrhœa, but never, under his notice, from local contusion, which Waller and Lee regard as the most common cause. We have seen cases occurring from all these causes, and two instances in which it followed the use of ergot, administered for the relief of menorrhagia.

The author mentions two interesting cases in which hysteritis followed an opening made through the imperforate hymen, for the discharge of retained menstrual fluid. In the first case, the operator, after evacuating "a considerable quantity of treacly fluid," injected warm water, in order to wash out all the collected secretion. Inflammation of the uterus followed in twelve hours, and the woman died. In the other case, "a small opening was made through the obstructing membrane, in order that the fluid might be gradually evacuated, and thus the chances of subsequent inflammation lessened; but, notwithstanding this precaution, urgent inflammatory symptoms, demanding prompt and active treatment, made their appearance."

The most important is the *third part*, or that which treats of malignant diseases, and especially of the varieties of cancer, of which he enumerates *three*, viz: "Encephaloid, Scirrhous, and Colloid." He regards cancer as constitutional from the beginning, and, consequently, even when the part is excised, the disease is liable to return and destroy the patient. Carcinomatous degenerations destroy the tissues of the part, whether bone, muscle, tendon or nerves. It seems not merely to change the natural structure of the part, but the peculiar structures of carcinoma are developed within and around them. Its development is usually accompanied with the firm adhesion of the parts to those surrounding it, so that it becomes more fixed and immoveable than other growths.

*Anatomical description of three varieties.* The *encephaloid* is characterised by dense septa, crossing the mass, and dividing it into lobes or lobules—the *scirrhous* variety has dense fibrous bands, deposited in lines either crossing each other, or radiating from a central cartilaginous nucleus, and enclosing a bluish, grey, or whitish matter—in the *colloid* variety the whole surface is divided into a great number of "distinct alveoli," regularly arranged, and of an oblong or oval shape; "the septa forming the walls of these being of a fibrous structure."

No case occurred to Dr. Lever of cancer being communicated by infection, although he met with instances of its hereditary transmission through several generations.

On most of the points of pathology, as well as descriptions of the diseases embraced in this work, we discover a very general concurrence between Dr. Lever and other late writers on the same subject, particularly Lee, Ashwell and Churchill; nor do we find any notable difference in the treatment recommended.

---

*Anatomical Atlas, illustrative of the Structure of the Human Body.* By Henry H. Smith, M. D. Part IV.

Already is this valuable work nearly completed. Part fourth, now issued, contains illustrations of the organs of Respiration and Circulation, making in all *ninety-eight figures.*

Part V., which will shortly appear, will complete the work. A few errors, we regret to observe, continue to occur. For example, while the figures are reduced copies of larger drawings, in several instances, the heading which precedes the reference has not been correspondingly altered. One or more of these examples we have observed in preceding publications, which perhaps led the author astray. Thus, Fig. 432, "*A view of the termination of the Arteries in the Veins as shown in the Web of a Frog's foot—magnified three diameters;*" and the next, or Fig. 433, which represents the capillary circulation of the same structure—"*magnified* 110 *diameters.*" These are undoubtedly errors. But there are some of the same character which strike the eye of a common observer more readily, and are, therefore, less liable to mislead, as in Fig. 449—"A view of the arteries of the neck and shoulder—*the size of life*,' although the whole figure is only about two inches by three and a half. Again, Fig. 465, "A view of the abdominal aorta and its branches—*the size of life.*"

We are perfectly aware of the difficulty, when passing rapidly through the press, of avoiding what may be regarded as minor errors, particularly in a first edition, and therefore merely wish to call the author's attention to the subject, that the proper corrections may be made in a future edition, which will, we have no doubt, be speedily called for.

The present, in all respects is equal to the numbers which have preceded it, and in mechanical execution it appears to us to be better than the last, or part third.

---

*Dissertation on the respect due to the Medical Profession, and the reason that it is not awarded by the community.* By Worthington Hooker, M. D. Read at the Annual Meeting of the Connecticut Medical Society, May 8th, 1844.

In this dissertation the author points out the causes which tend to degrade the profession; first, as it regards the conduct of those not of the profession; and especially members of the other learned professions: secondly, the causes which originate in the misconduct of its own members: lastly, he points out what he believes to be the proper remedies. These consist essentially in a rigid observance of the laws of ethics. The dissertation is a sensible and well written production, every way creditable to the author.

---

*Cyclopædia of Practical Medicine.*

Already we have part *XII*, which completes the first half of the work, and consequently concludes the second volume. The last article in this *part* is on inflammation, and is written by Drs. Crawford and Tweedie.

